# Sodium fluoride causes hepatocellular S-phase arrest by activating ATM-p53-p21 and ATR-Chk1-Cdc25A pathways in mice

**DOI:** 10.18632/oncotarget.23093

**Published:** 2017-12-11

**Authors:** Huan Liu, Qin Luo, Hengmin Cui, Huidan Deng, Ping Kuang, Yujiao Lu, Jing Fang, Zhicai Zuo, Junliang Deng, Yinglun Li, Xun Wang, Ling Zhao

**Affiliations:** ^1^ College of Veterinary Medicine, Sichuan Agricultural University, Wenjiang, Chengdu, China; ^2^ Key Laboratory of Animal Diseases and Environmental Hazards of Sichuan Province, Sichuan Agriculture University, Wenjiang, Chengdu, China

**Keywords:** NaF, S phase arrest, protein expression, mRNA expression, liver, Immunology and Microbiology Section, Immune response, Immunity

## Abstract

In this study, experimental pathology, flow cytometry (FCM), quantitative real-time polymerase chain reaction (qRT-PCR), and western blot (WB) were used to evaluate the effects of sodium fluoride (NaF) on hepatocellular cell cycle progression in mice. A total of 240 ICR mice were divided equally into four groups; the experimental groups received 12, 24, or 48 mg/kg NaF intragastrically for 42 days, while the control group received distilled water. Doses of NaF above 12 mg/kg increased the percentage of cells in S phase (S-phase arrest), reduced percentages of cells in G0/G1 or G2/M phase, and activated the ATM-p53-p21 and ATR-Chk1-Cdc25A pathways. Activation of these pathways was characterized by up-regulation of ATM, p53, p21, ATR, and Chk1 mRNA and protein expression, and down-regulation of Cdc25A, cyclin E, cyclin A, CDK2, CDK4, and proliferating cell nuclear antigen (PCNA) mRNA and protein expression. These results indicate that NaF caused S-phase arrest by activating the ATM-p53-p21 and ATR-Chk1-Cdc25A pathways.

## INTRODUCTION

Although fluorine is an essential mineral element for all mammals, including humans, exposure to high fluorine levels has toxic effects on various organs and tissues [1]. Fluorine, which is added to drinking water, is rapidly absorbed by the gastrointestinal tract after ingestion [2]. Other common sources of fluorine include dental products, food, drugs, and industrial emissions [3]. Excessive fluoride intake can have many harmful effects, including damage to the teeth, bones, and other organs [4]. We have previously demonstrated that excessive fluorine intake can induce cytotoxicity, immunotoxicity, oxidative damage, and pathological injury in the thymus [5], spleen [6–8], bursa of Fabricius [9], cecal tonsil [10–14], liver [15, 16], kidney [17–19], peripheral blood [20–23], and intestine [24–28] in broiler chickens. Other studies have also demonstrated that fluoride induces cytotoxicity, apoptosis, and DNA damage in both humans and animals [1, 29–31].

Dysregulation of the cell cycle, which is crucial for the maintenance of homeostasis in multicellular organisms [32], may result in uncontrolled cell proliferation or excessive cell death; such changes can promote tumor formation and various other disease states [32–34]. DNA damage activates molecules that inhibit the cell cycle and promote cell death to prevent proliferation of genetically altered cells [32]. Excessive fluoride increases the G0/G1 cell cycle phase population in thymocytes and splenocytes [5, 8] and reduces numbers of T and B cells in young broiler chickens. However, fluoride increases the S phase and decreases the G2/M phase population in rat osteoblasts, but does not affect the size of the G0/G1 phase population [34]. We recently demonstrated that sodium fluoride (NaF) inhibits proliferation and induces apoptosis in mouse splenic lymphocytes both *in vivo* and *in vitro* [3, 8, 35, 36] and increases G0/G1 arrest in the broiler chicken kidney [17] and thymus [5]. Furthermore, the molecular mechanisms underlying the ability of NaF to inhibit proliferation in splenic lymphocytes include alterations in the expression of cytokine proteins and increases in cell cycle arrest [36]. The liver is a crucial metabolic organ that regulates the metabolism of trace elements, and excessive fluoride intake can also damage hepatic tissue. We have previously demonstrated that elevated fluorine levels result in oxidative damage, pathological injuries [16], and S phase arrest [37] in the livers of broiler chickens. Shashi *et al*. [2] reported that fluoride exposure activated hepatic enzymes during osteofluorosis. In addition, fluoride caused lesions and induced the synthesis of stress proteins in the livers and kidneys of mice [38–40]. Chen *et al*. [41] reported that excessive fluoride inhibited hepatocellular proliferation and differentiation, which might be related to increased S-phase arrest.

Although some studies have examined fluorine-induced cell cycle arrest, very few have examined this relationship in liver cells either *in vivo* or *in vitro*, and the mechanisms underlying fluoride-induced cell cycle arrest in the liver remain unclear. In this study, we examined hepatocellular cell cycle distribution and cell cycle regulatory protein levels in mice to determine how NaF suppresses hepatocellular proliferation. After the administration of different concentrations of NaF, hepatocellular cell cycle distribution and the expression of cell cycle control molecules, including phosphorylated ataxia-telangiectasia-mutated (p-ATM, Ser1981), phosphorylated ATM- and Rad3-related (p-ATR, Thr1989), p-p53(Ser15), p21, p-Chk1(Ser317), p-Cdc25A(Ser76), cyclin E/D/A/B, CDK1/2/4, and proliferating cell nuclear antigen (PCNA) were examined using flow cytometry (FCM), western blot (WB), and quantitative real-time polymerase chain reaction (qRT-PCR).

## RESULTS

### Fluoride inhibited liver development

**Figure 1:**
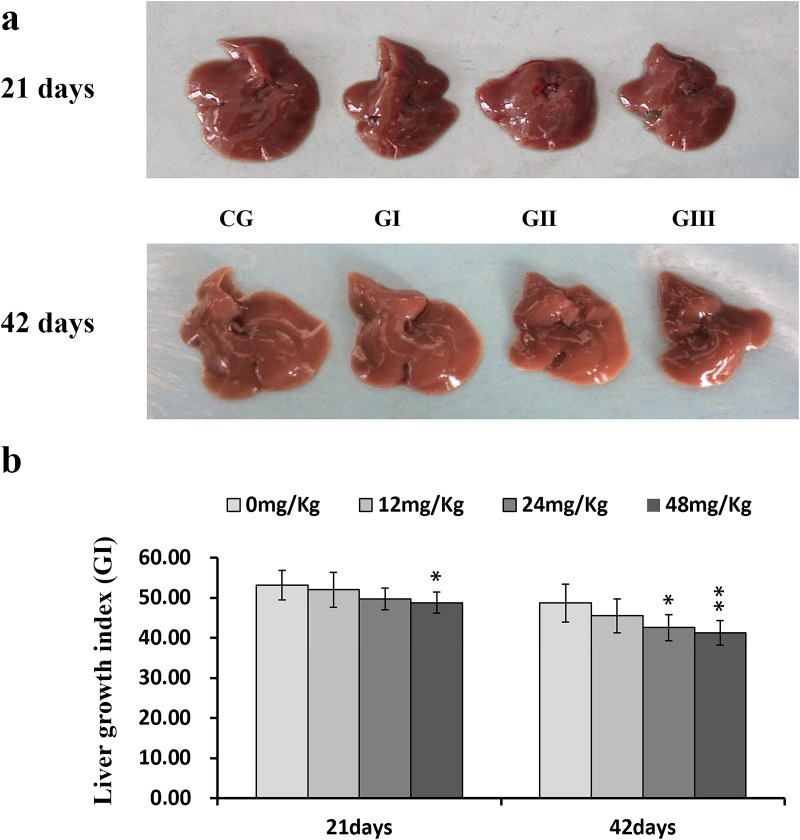
Changes of the liver (a) and changes of the growth index (GI, b) of liver at 21 and 42 days of experiment Livers are smaller in the three NaF-treated groups than those in the control group. Hepatic growth index is decreased in the 24 and 48 mg/kg groups. CG: Control group; GI: 12mg/kg group; GII: 24mg/kg group; GIII: 48mg/kg group. b changes of the growth index (GI) in liver, *P<0.05, compared with the control group **P<0.01, compared with the control group.

Liver development was evaluated based on liver growth index (GI) values. GI values were similar in the control and 12 mg/kg groups throughout the 42-day experiment and in the control and 24 mg/kg groups on day 21 of the experiment. However, GI was lower (*P* < 0.05 and *P* < 0.01) in the 48 mg/kg group on days 21 and 42 and in the 24 mg/kg group on day 42 than in the control group (Figure 1b).

### Fluoride induced pathological changes in the liver

Macroscopically, livers were smaller in the three NaF treatment groups than in the control group (Figure 1a). However, there were no obvious changes in hepatic color or texture.

**Figure 2:**
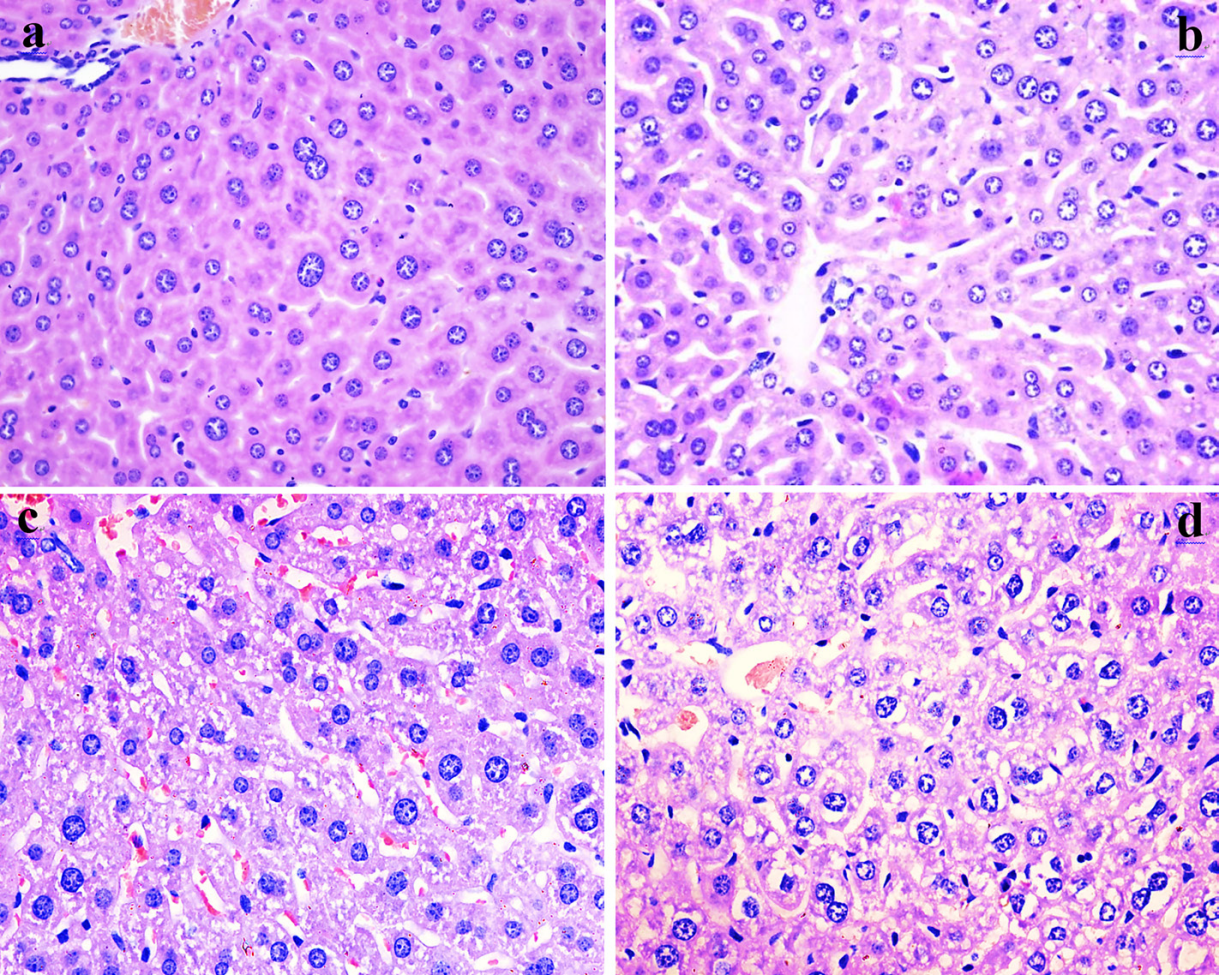
Histopathological changes in the liver at 21 days of experiment (**a**) The control group (H&E ×400). (**b**) The 12 mg/kg group. Hepatocytes show slight granular and vacuolar degeneration (H&E ×400). (**c**) The 24 mg/kg group. Hepatocytes show granular and vacuolar degeneration (H&E ×400). (**d**) The 48 mg/kg group. Hepatocytes show obvious granular and vacuolar degeneration. Also, necrotic hepatocytes are observed (H&E ×400).

**Figure 3:**
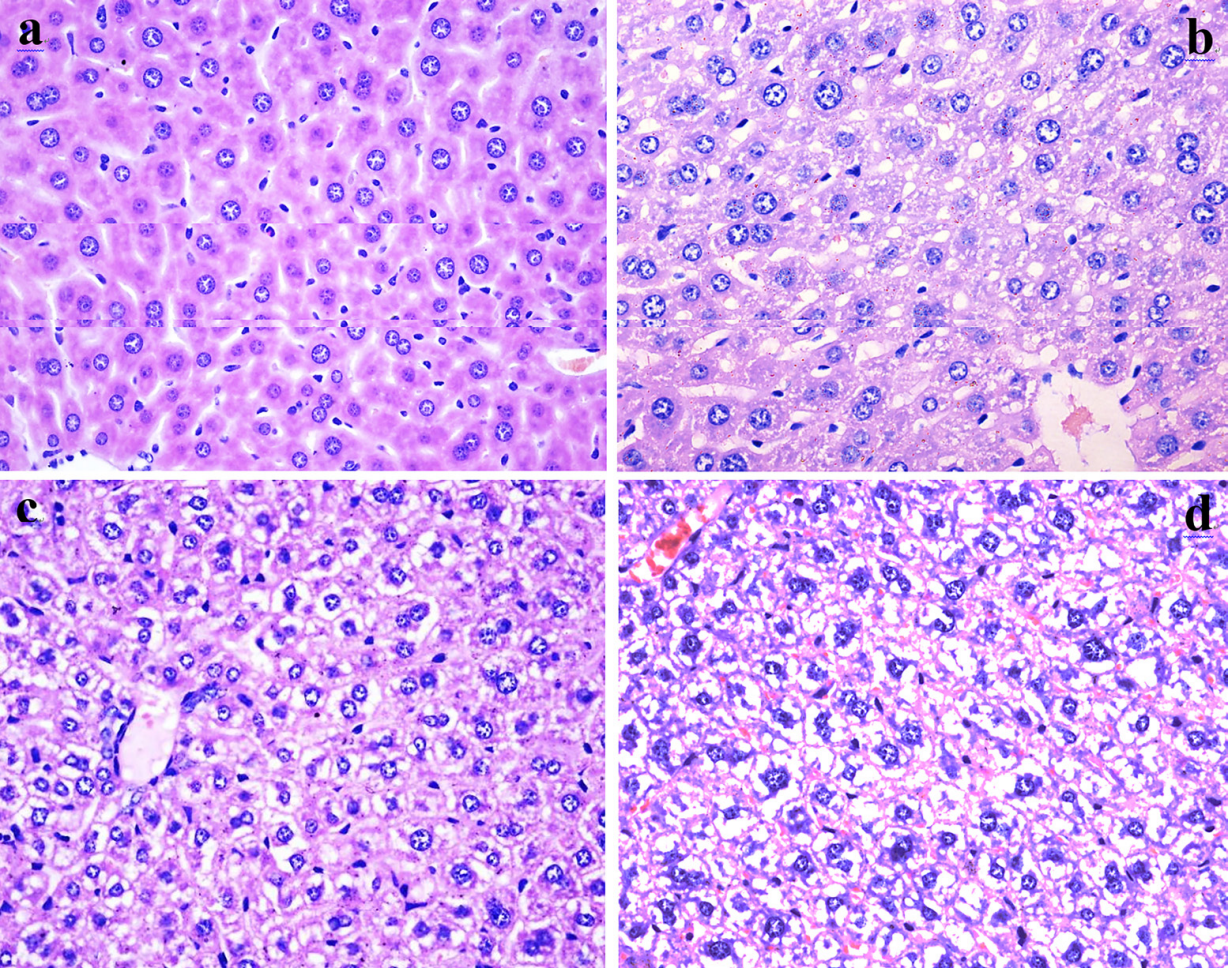
Histopathological changes in the liver at 42 days of experiment (**a**) The control group (H&E ×400). (**b**) The 12 mg/kg group. Hepatocytes show granular and vacuolar degeneration (H&E ×400). (**c**) The 24 mg/kg group. Hepatocytes show obvious granular and vacuolar degeneration (H&E ×400). (**d**) The 48 mg/kg group. Hepatocytes show significant granular and vacuolar degeneration. Also, Necrotic hepatic cells are observed (H&E ×400).

As shown in Figures 2 and 3, fluoride administration increased the number of hepatocytes with granular and vacuolar degeneration in a dose- and time-dependent manner, and tiny particles as well as small or large vacuoles were visible in the cytoplasm of these hepatocytes. Necrotic hepatocytes were also observed in the 48 mg/kg group. There was no evidence of these lesions in the control group.

### NaF induced S-phase arrest in the liver

**Figure 4:**
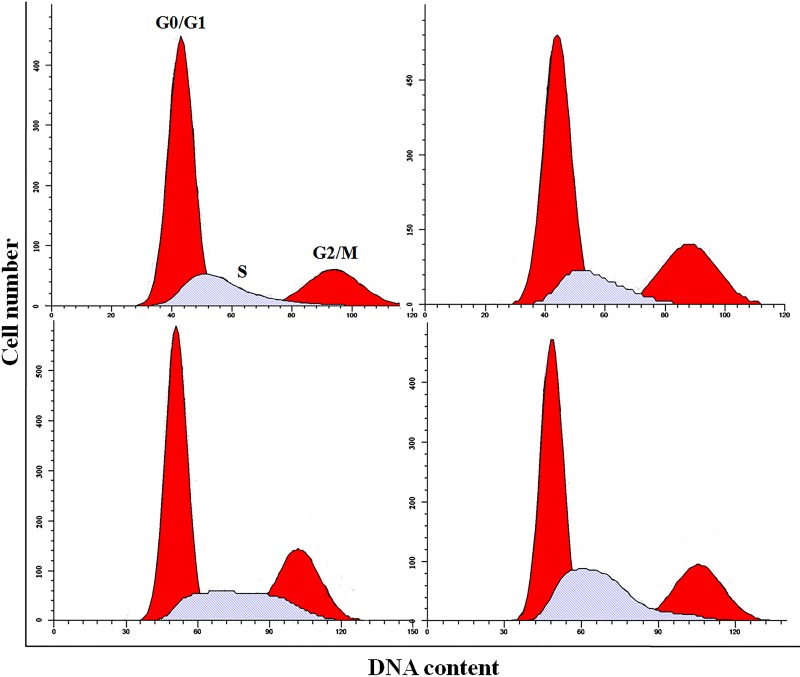
Cell cycle changes in the liver at 21 days of age by flow cytometry

**Figure 5:**
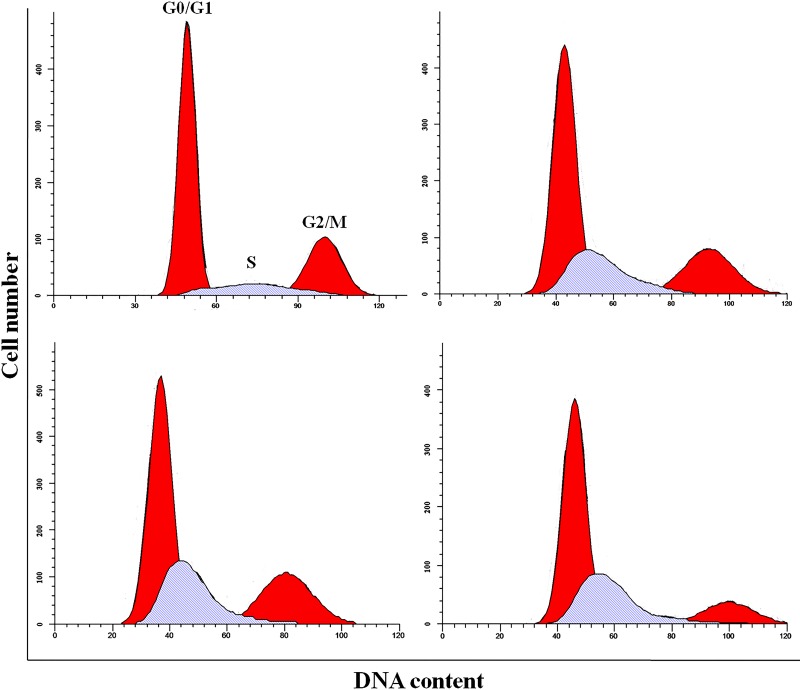
Cell cycle changes in the liver at 42 days of age by flow cytometry

**Figure 6:**
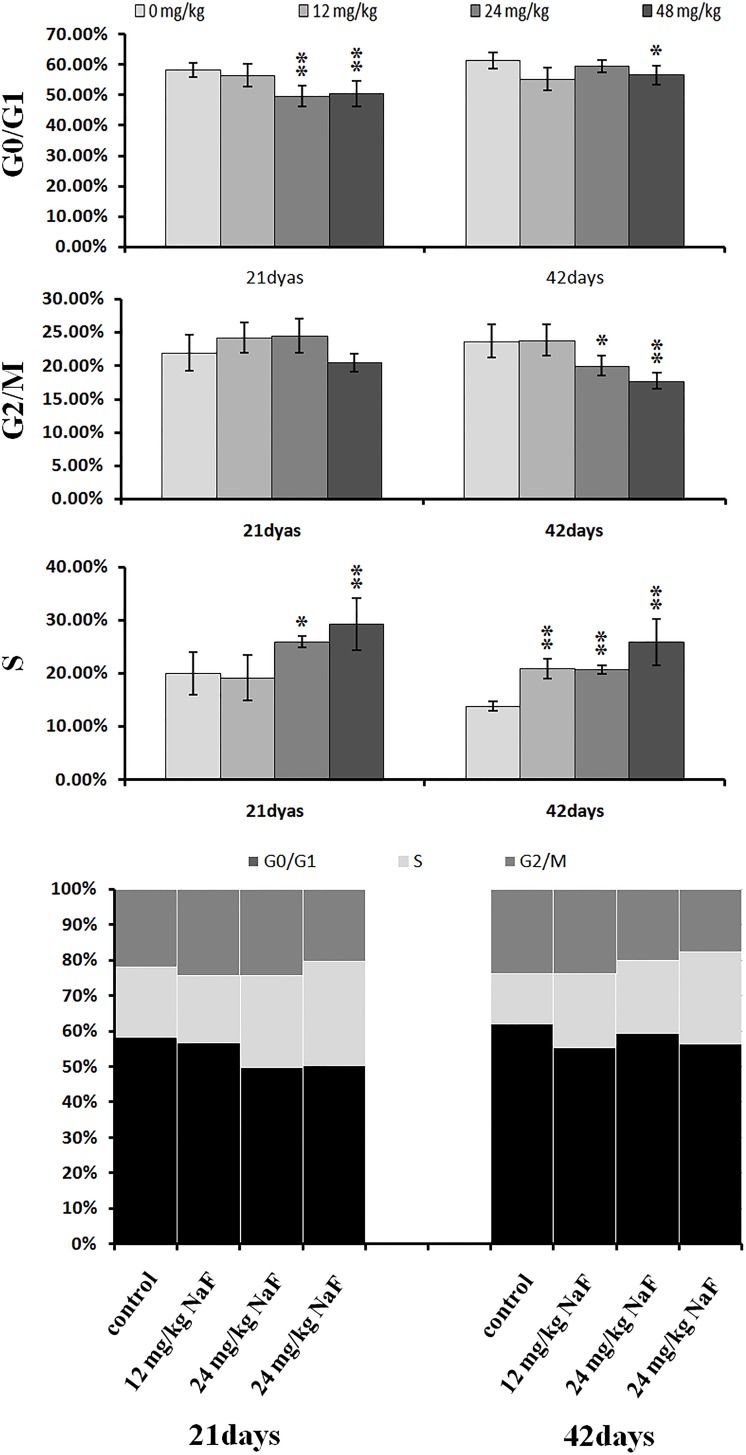
Changes of cell cycle phase distribution (%) in the liver Data are presented with the mean ± standard deviation (n=8),*P<0.05, compared with the control group **P<0.01, compared with the control group.

As shown in Figures 4-6, percentages of hepatocytes in S phase increased in a dose- and time-dependent manner, while percentages of hepatocytes in the G0/G1 and G2/M phases decreased.

Percentages of cells in the G0/G1 phase decreased (*p* < 0.05 or *p* < 0.01) in the 48 mg/kg group at both days 21 and 42 and in the 24 mg/kg group at day 21 of the experiment when compared to the control group. Percentages of cells in the G2/M phase were also lower (*p* < 0.05 or *p* < 0.01) in the 24 mg/kg and 48 mg/kg groups at day 42 of the experiment than in the control group. Finally, percentages of cells in S phase increased (*p* < 0.05 or *p* < 0.01) in the 24 mg/kg and 48 mg/kg groups at days 21 and 42 and in the 12 mg/kg group at day 42 of the experiment when compared to the control group.

### Changes in expression of cyclins, CDKs, and PCNA in the liver

To understand the mechanisms underlying NaF-induced hepatocyte cell cycle arrest, we evaluated the expression of regulatory molecules associated with the G1/S phase, including cyclin E/D/A/B, CDK1/2/4, and PCNA, at the protein and mRNA levels. The results are shown in Figures 7-14.

**Figure 7:**
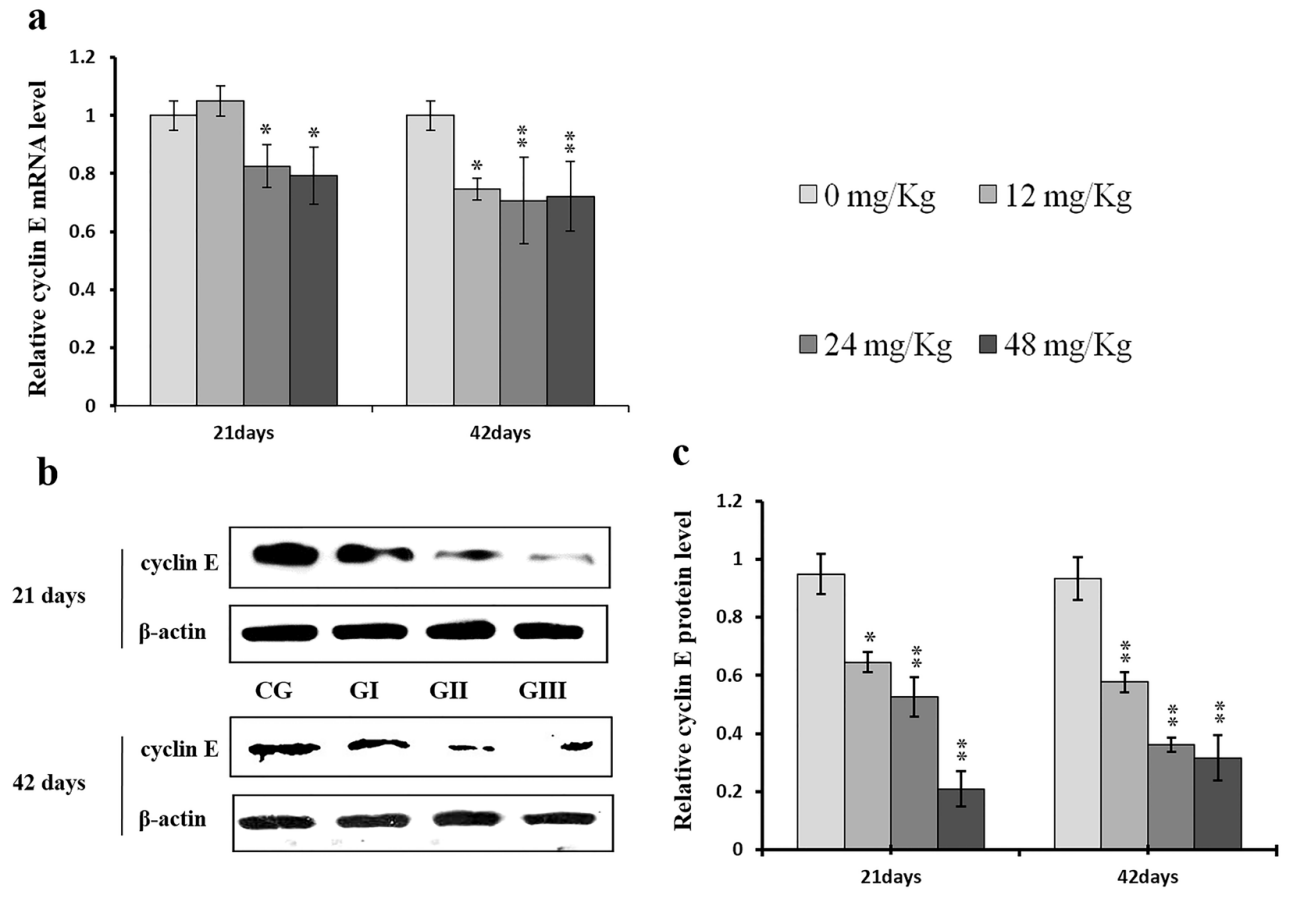
Changes of mRNA and protein expression levels of cyclin E in the liver at 21 and 42 days of experiment (**a)** The relative mRNA expression levels of cyclin E. (**b**) The western blot assay of cyclin E. (**c**) The relative protein expression levels of cyclin E. CG: Control group; GI: 12mg/kg group; GII: 24mg/kg group; GIII: 48mg/kg group. Data are presented with the means ± standard deviation (n=8), *p<0.05, compared with the control group; **p<0.01, compared with the control group.

**Figure 8:**
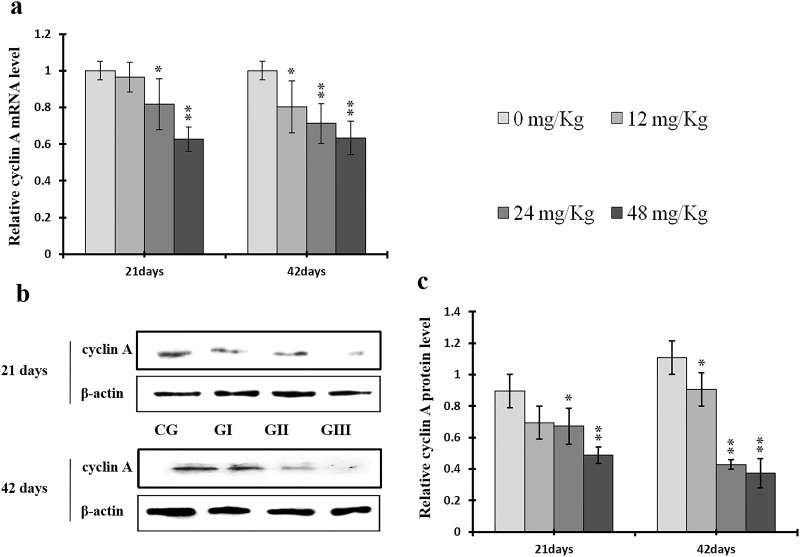
Changes of mRNA and protein expression levels of cyclin A in the liver at 21 and 42 days of experiment (**a**) The western blot assay of cyclin A. (**b**) The relative mRNA expression levels of cyclin A. (**c**) The relative protein expression levels of cyclin A. CG: Control group; GI: 12mg/kg group; GII: 24mg/kg group; GIII: 48mg/kg group. Data are presented with the means ± standard deviation (n=8), *p<0.05, compared with the control group; **p<0.01, compared with the control group.

**Figure 9:**
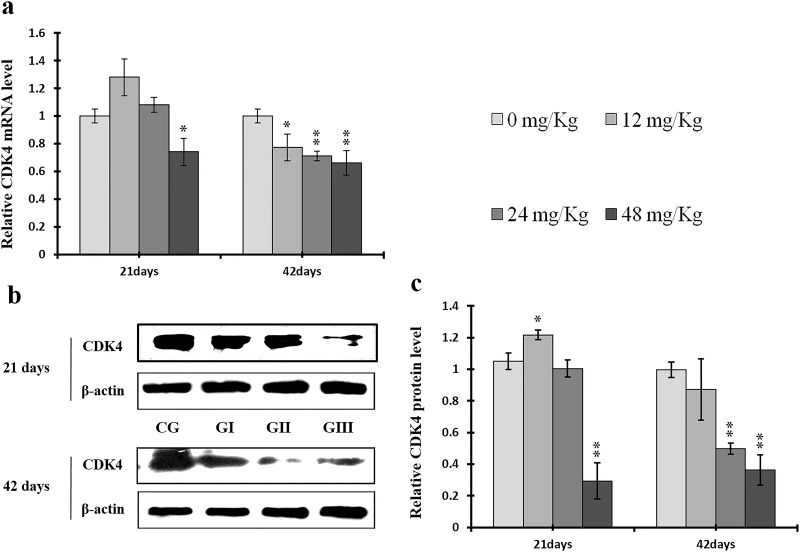
Changes of mRNA and protein expression levels of CDK4 in the liver at 21 and 42 days of experiment (**a**) The relative mRNA expression levels of CDK4. (**b**) The western blot assay of CDK4. (**c**) The relative protein expression levels of CDK4. CG: Control group; GI: 12mg/kg group; GII: 24mg/kg group; GIII: 48mg/kg group. Data are presented with the means ± standard deviation (n=8), *p<0.05, compared with the control group; **p<0.01, compared with the control group.

**Figure 10:**
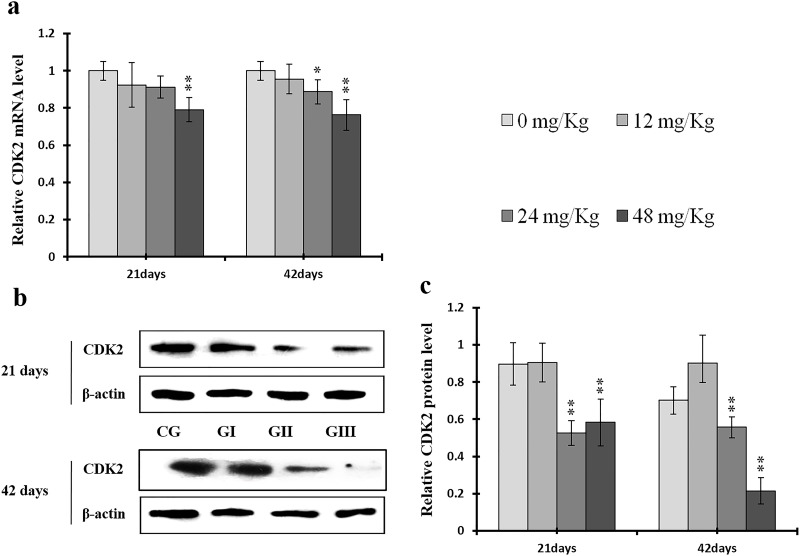
Changes of mRNA and protein expression levels of CDK2 in the liver at 21 and 42 days of experiment (**a**) The relative mRNA expression levels of CDK2. (**b**) The western blot assay of CDK2. (**c**) The relative protein expression levels of CDK2. CG: Control group; GI: 12mg/kg group; GII: 24mg/kg group; GIII: 48mg/kg group. Data are presented with the means ± standard deviation (n=8), *p<0.05, compared with the control group; **p<0.01, compared with the control group.

**Figure 11:**
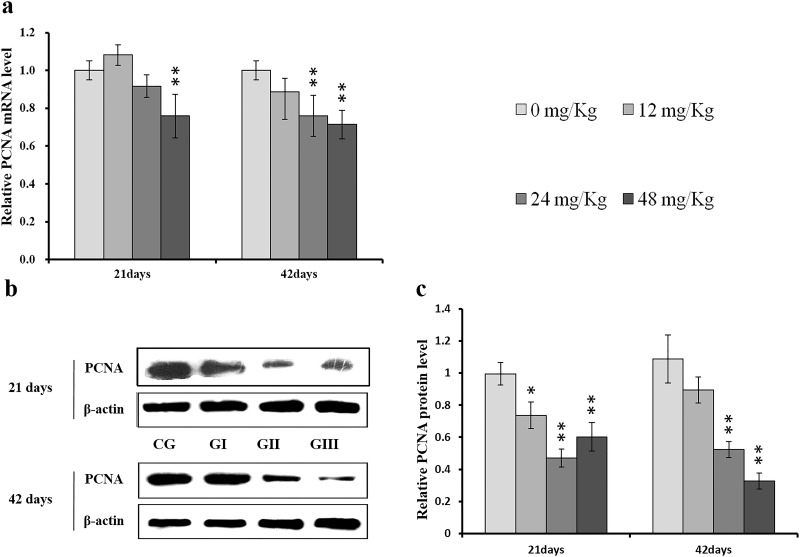
Changes of mRNA and protein expression levels of PCNA in the liver at 21 and 42 days of experiment (**a**) The relative mRNA expression levels of PCNA. (**b**) The western blot assay of PCNA. (**c**) The relative protein expression levels of PCNA. CG: Control group; GI: 12mg/kg group; GII: 24mg/kg group; GIII: 48mg/kg group. Data are presented with the means ± standard deviation (n=8), *p<0.05, compared with the control group; **p<0.01, compared with the control group.

**Figure 12:**
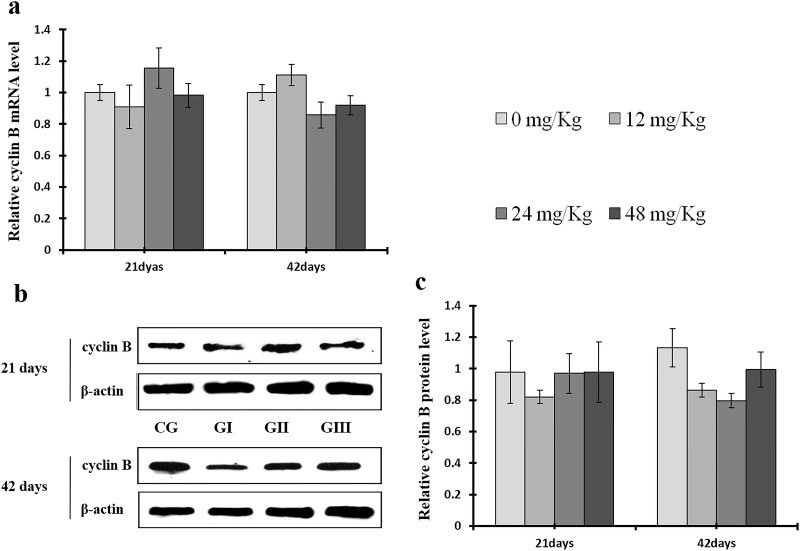
Changes of mRNA and protein expression levels of cyclin B in the liver at 21 and 42 days of experiment (**a**) The relative mRNA expression levels of cyclin B. (**b**) The western blot assay of cyclin B. (**c**) The relative protein expression levels of cyclin B. CG: Control group; GI: 12mg/kg group; GII: 24mg/kg group; GIII: 48mg/kg group. Data are presented with the means ± standard deviation (n=8), *p<0.05, compared with the control group; **p<0.01, compared with the control group.

**Figure 13:**
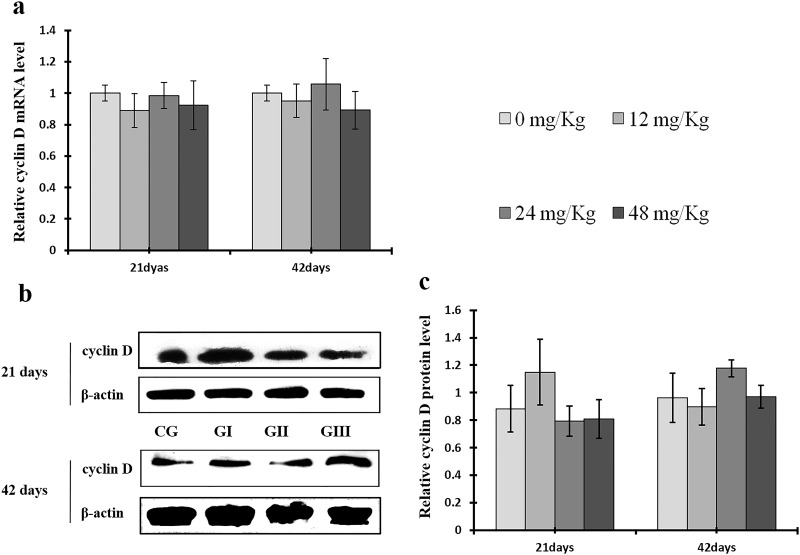
Changes of mRNA and protein expression levels of cyclin D in the liver at 21 and 42 days of experiment (**a**) The relative mRNA expression levels of cyclin D. (**b**) The western blot assay of cyclin D. (**c**) The relative protein expression levels of cyclin D. CG: Control group; GI: 12mg/kg group; GII: 24mg/kg group; GIII: 48mg/kg group. Data are presented with the means ± standard deviation (n=8), *p<0.05, compared with the control group; **p<0.01, compared with the control group.

**Figure 14:**
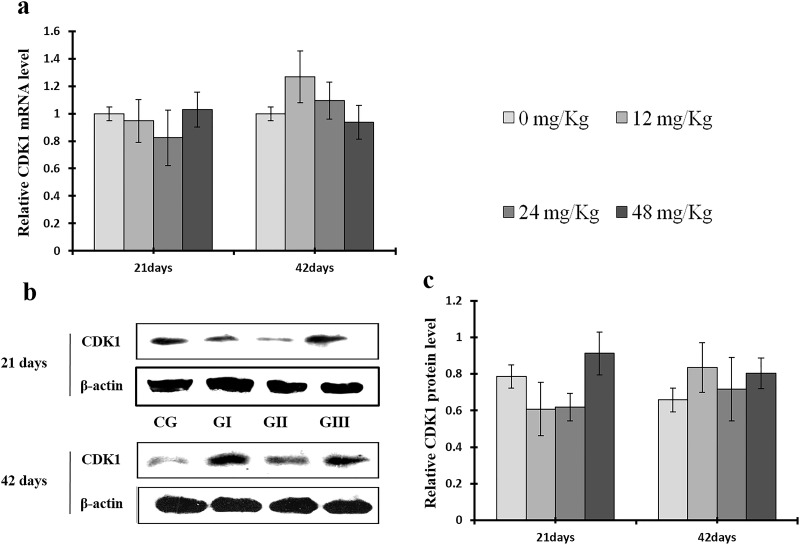
Changes of mRNA and protein expression levels of CDK1 in the liver at 21 and 42 days of experiment (**a**) The relative mRNA expression levels of CDK1. (**b**) The western blot assay of CDK1. (**c**) The relative protein expression levels of CDK1. CG: Control group; GI: 12mg/kg group; GII: 24mg/kg group; GIII: 48mg/kg group. Data are presented with the means ± standard deviation (n=8), *p<0.05, compared with the control group; **p<0.01, compared with the control group

Cyclin E and A protein levels were decreased (*p* < 0.05 or *p* < 0.01) in the 24 mg/kg and 48 mg/kg groups at day 21 and in all three NaF-treated groups at day 42 of the experiment when compared to the control group; cyclin E protein levels were also decreased (*p* < 0.05) in the 12 mg/kg group at day 21 of experiment in comparison to the control group (Figures 7c and 8c). Similarly, cyclin E and A mRNA expression was lower (*p* < 0.05 or *p* < 0.01) in the 24 mg/kg and 48 mg/kg groups at days 21 and 42 and in the 12 mg/kg group at day 42 of the experiment than in the control group (Figures 7a and 8a).

As shown in Figure 9c, CDK4 protein levels were decreased (*p* < 0.05 or *p* < 0.01) in the 48 mg/kg group at days 21 and 42 and in the 24 mg/kg group at day 42 of the experiment, but were increased in the 12 mg/kg group at day 21 of the experiment, when compared to the control group. CDK2 protein levels were also lower (*p* < 0.05 or *p* < 0.01) in the 24 mg/kg and 48 mg/kg groups at days 21 and 42 and in the 12 mg/kg group at day 42 of the experiment than in the control group (Figure 10c). CDK4 mRNA expression was similarly decreased (*p* < 0.05 or *p* < 0.01) in the 48 mg/kg group at days 21 and 42 and in the 12 mg/kg and 24 mg/kg groups at day 42 of the experiment (Figure 9a). CDK2 mRNA expression was also lower (*p* < 0.05 or *p* < 0.01) in the 48 mg/kg group at days 21 and 42 and in the 24 mg/kg group at day 42 of the experiment than in the control group (Figure 10a). PCNA protein levels were decreased (*p* < 0.05 or *p* < 0.01) in the 24 mg/kg and 48 mg/kg groups at days 21 and 42 and in the 12 mg/kg group at day 21 of the experiment when compared to the control group. PCNA mRNA expression was also lower (*p* < 0.05 or *p* < 0.01) in the 48 mg/kg group at days 21 and 42 and in the 24 mg/kg group at day 42 of experiment than in the control group (Figure 11). In contrast, cyclin B/D and CDK1 protein and mRNA expression did not differ among any of the groups (Figures 12-14).

### Changes in protein and mRNA expression of cyclin/CDK regulators in the liver

**Figure 15:**
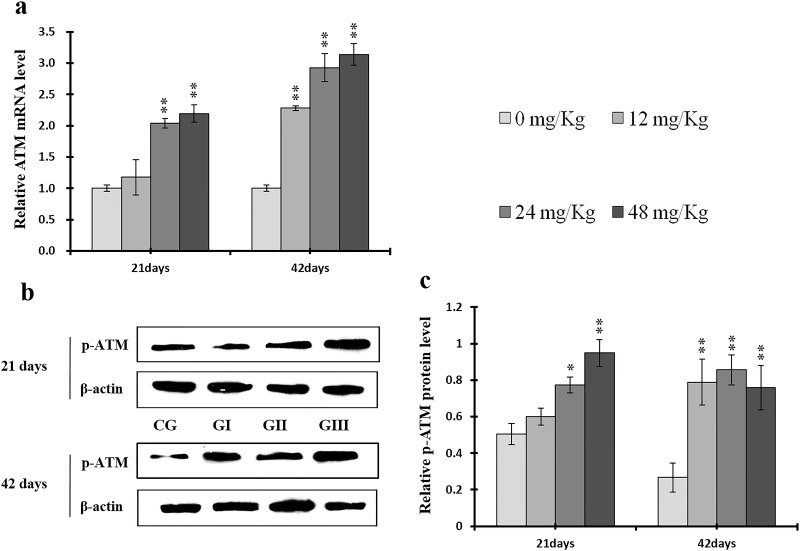
Changes of mRNA level of ATM and protein expression levels of p-ATM in the liver at 21 and 42 days of experiment (**a**) The relative mRNA expression levels of ATM. (**b**) The western blot assay of p-ATM. (**c**) The relative protein expression levels of p-ATM. CG: Control group; GI: 12mg/kg group; GII: 24mg/kg group; GIII: 48mg/kg group. Data are presented with the means ± standard deviation (n=8), *p<0.05, compared with the control group; **p<0.01, compared with the control group.

**Figure 16:**
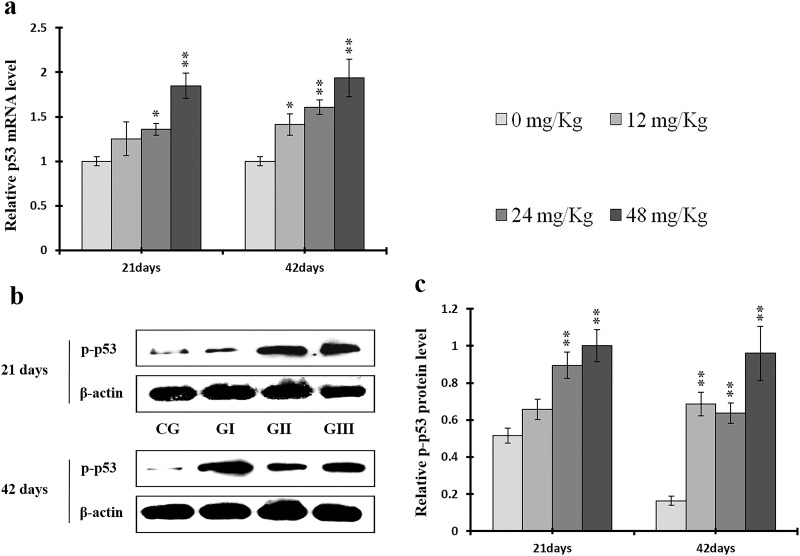
Changes of mRNA level of p53 and protein expression levels of p-p53 in the liver at 21 and 42 days of experiment (**a**) The relative mRNA expression levels of p53. (**b**) The western blot assay of p-p53. (**c**) The relative protein expression levels of p-p53.CG: Control group; GI: 12mg/kg group; GII: 24mg/kg group; GIII: 48mg/kg group. Data are presented with the means ± standard deviation (n=8), *p<0.05, compared with the control group; **p<0.01, compared with the control group.

**Figure 17:**
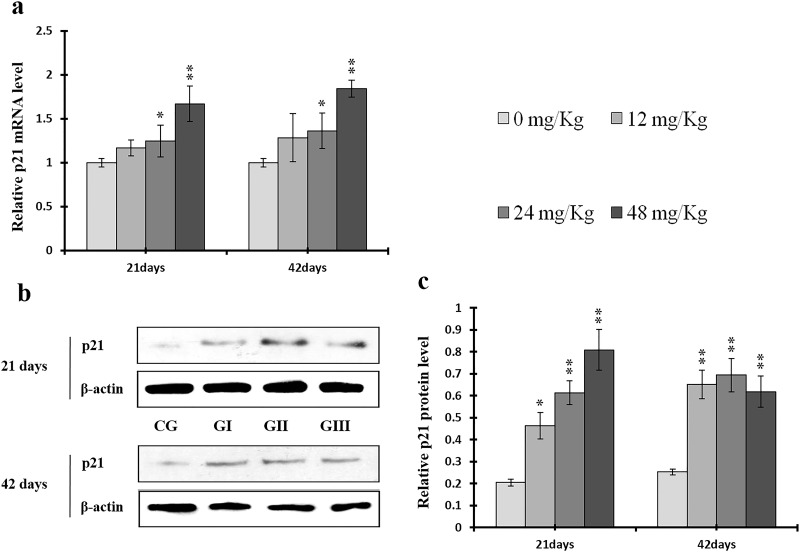
Changes of mRNA and protein expression levels of p21 in the liver at 21 and 42 days of experiment (**a**) The relative mRNA expression levels of p21. (**b**) The western blot assay of p21. (**c**) The relative protein expression levels of p21. CG: Control group; GI: 12mg/kg group; GII: 24mg/kg group; GIII: 48mg/kg group. Data are presented with the means ± standard deviation (n=8), *p<0.05, compared with the control group; **p<0.01, compared with the control group.

**Figure 18:**
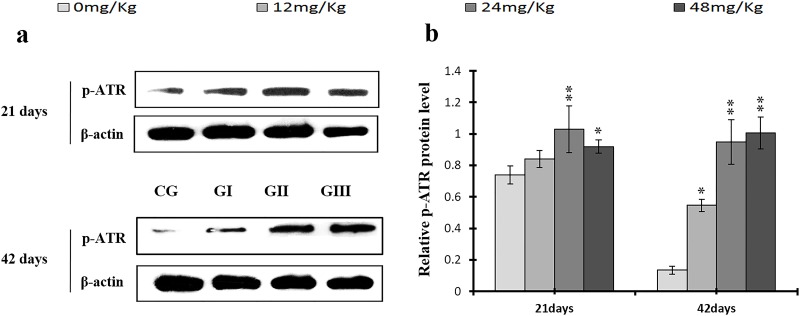
Changes of protein expression levels of p-ATR in the liver at 21 and 42 days of experiment (**a**) The western blot assay of p-ATR. (**b**) The relative protein expression levels of p-ATR. CG: Control group; GI: 12mg/kg group; GII: 24mg/kg group; GIII: 48mg/kg group. Data are presented with the means ± standard deviation (n=8), *p<0.05, compared with the control group; **p<0.01, compared with the control group.

**Figure 19:**
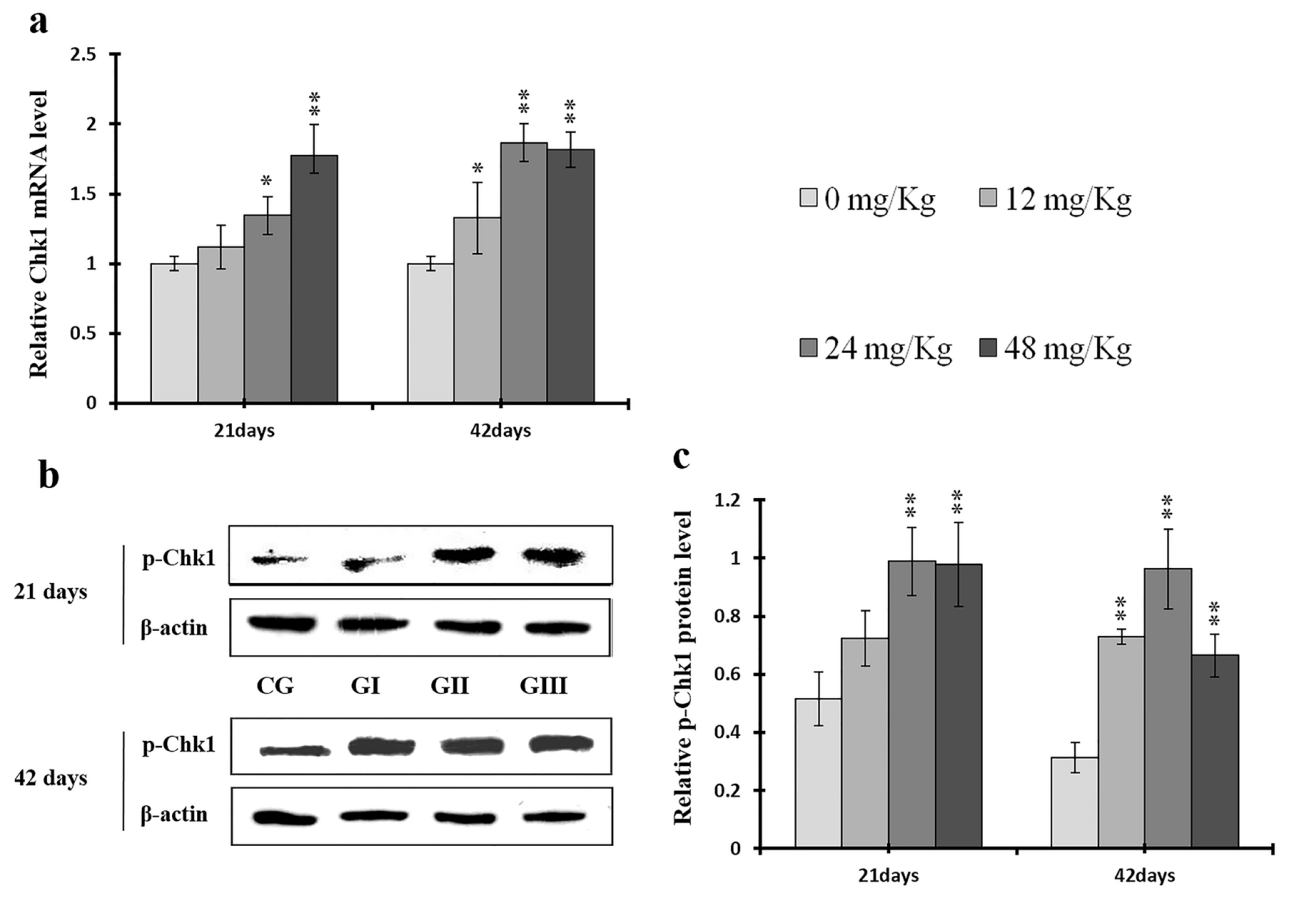
Changes of mRNA level of Chk1 and protein expression levels of p-Chk1 in the liver at 21 and 42 days of experiment (**a**) The relative mRNA expression levels of Chk1. (**b**) The western blot assay of p-Chk1. c The relative protein expression levels of p-Chk1. CG: Control group; GI: 12mg/kg group; GII: 24mg/kg group; GIII: 48mg/kg group. Data are presented with the means ± standard deviation (n=8), *p<0.05, compared with the control group; **p<0.01, compared with the control group.

**Figure 20:**
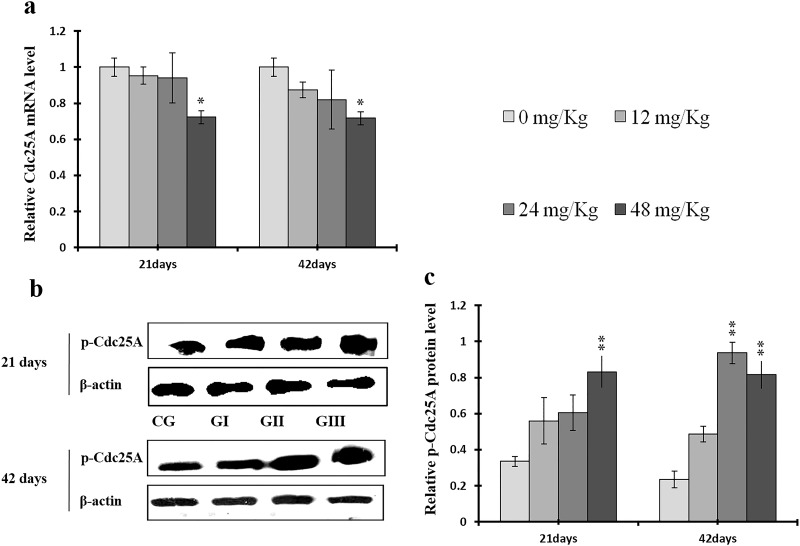
Changes of mRNA level of Cdc25A and protein expression levels of p-Cdc25A in the liver at 21 and 42 days of experiment (**a**) The relative mRNA expression levels of Cdc25A. (**b**) The western blot assay of p-Cdc25A. (**c**) The relative protein expression levels of p-Cdc25A. CG: Control group; GI: 12mg/kg group; GII: 24mg/kg group; GIII: 48mg/kg group. Data are presented with the means ± standard deviation (n=8), *p<0.05, compared with the control group; **p<0.01, compared with the control group.

Cdc25A promotes cell cycle progression by activating cyclin-dependent protein kinases, and its production is associated with entry of cells into the S phase [42–44]. Moreover, exposure to novel pactamycin analogs also triggers p53-dependent S-phase cell cycle arrest in human head and neck squamous cell carcinoma (HNSCC) [45]. To determine whether the Cdc25A and p53 pathway is involved in NaF-induced cell cycle arrest, we examined the expression of ATR-Chk1-Cdc25A and ATM-p53-p21 pathway members at both the protein and mRNA levels [43, 46, 47]. The results are shown in Figures 15-20.

P-ATM protein and ATM mRNA levels were increased (*p* < 0.05 or *p* < 0.01) in the 24 mg/kg and 48 mg/kg groups at days 21 and 42 and in the 12 mg/kg group at day 42 of the experiment when compared to the control group (Figure 15). P-p53 protein and p53 mRNA levels were also increased (*p* < 0.05 or *p* < 0.01) in the 24 mg/kg and 48 mg/kg groups at days 21 and 42 and in the 12 mg/kg group at day 42 of the experiment in comparison to the control group (Figure 16). P21 protein levels were higher (*p* < 0.05 or *p* < 0.01) in all three NaF-treated groups than in the control group at days 21 and 42 of the experiment. Similarly, p21 mRNA expression was markedly increased (*p* < 0.05 or *p* < 0.01) in the 24 mg/kg and 48 mg/kg groups at days 21 and 42 of the experiment when compared to the control group (Figure 17).

As shown in Figures 18c and 19c, p-ATR and p-Chk1 protein levels were increased (*p* < 0.05 or *p* < 0.01) in the 24 mg/kg and 48 mg/kg groups at days 21 and 42 and in the 12 mg/kg group at day 42 of the experiment when compared to the control group. P-Chk1 mRNA expression was also markedly higher (*p* < 0.05 or *p* < 0.01) in the 24 mg/kg and 48 mg/kg groups at days 21 and 42 and in the 12 mg/kg group at day 42 of the experiment than in the control group (Figure 19a). NaF treatment increased (*p* < 0.05 or *p* < 0.01) p-Cdc25A protein levels in the 48 mg/kg group at days 21 and 42 and in the 24 mg/kg group at day 42 of the experiment in comparison to the control group. However, Cdc25A mRNA expression was decreased (*p* < 0.05 or *p* < 0.01) in the 48 mg/kg group at days 21 and 42 of the experiment when compared to the control group (Figure 20).

## DISCUSSION

In this study, we examined the molecular pathways underlying NaF-induced cell cycle arrest in the mouse liver. We found that, at does above 12 mg/kg, NaF induced cell cycle arrest in the S phase and decreased the percentages of cells in the G0/G1and G2/M phases (Figure 6).

Hayashi *et al*. [48] reported that NaF induced cell cycle-dependent cytotoxicity and clastogenicity in human diploid fibroblasts, and cells were especially sensitive to these effects during early and middle S phase. However, Aardema *et al*. [49] reported that NaF induced cell cycle was arrest in the G2/M phase and that cells in both the S and G2 phases were equally sensitive to the adverse effects of NaF [50]. Our results are consistent with those of Zhang *et al*. [51], who found that fluoride increased the proportion of cells in the S phase, decreased the proportions in both the G0/G1 and G2/M phases, and induced DNA damage in cultured primary cultured rat hippocampal neurons. Excessive fluoride levels can also increase the proportion of cells in the S phase, thereby affecting hepatocellular proliferation and differentiation [41].

Here, we examined the expression of S phase regulators to identify the molecular mechanisms underlying NaF-induced cell cycle arrest in the S phase in the liver. NaF increased p-ATM, p-p53, p21, p-ATR, p-Chk1, and p-Cdc25A protein levels, increased ATM, p53, p21, and Chk1 mRNA expression, and decreased Cdc25A mRNA expression (Figures 15-20). Li *et al*. [52] reported that fluoride induced p53 expression and cell cycle arrest in the S phase in human embryo hepatocytes in a dose-dependent manner; fluoride also increased or induced p53 expression in rat leukocytes [53] and human embryonic hepatocytes [54]. ATM plays a prominent role in checkpoint regulation at S phase transition and is associated with p53 phosphorylation after DNA damage-induced stresses [55–58]. Our results demonstrate conclusively that NaF up-regulates ATM and its downstream target p53, thus activating the ATM signal transduction pathways (Figures 15 and 16). These findings are similar to those of Guha *et al*., who demonstrated that novel pactamycin analogs can cause S-phase arrest in human head and neck squamous cell carcinoma (HNSCC) cells by increasing p53 activity, up-regulating expression of the cyclin kinase inhibitors p27 and p21, slightly reducing cyclin D1 expression, and moderately increasing cyclin E expression; no changes were observed in the expression of cyclin B, CDK2, or CDK4 [45]. Treatment with the novel anthraquinone derivative IMP1338 also increased p53-dependent cell cycle arrest in the S phase in human cancer cells [59]. P53 therefore may be a critical mediator of apoptosis, DNA repair, and cell cycle arrest in response to DNA damage and cellular stress [60]. In addition, p53 can up-regulate p21 expression, which also promotes cell cycle arrest in the S phase [61]. Here, NaF increased p21 protein levels and mRNA expression (Figure 17), which is consistent with studies demonstrating that various stimuli induce cell cycle arrest in the S phase through p53-dependent activation of p21 [45, 62–64]. P21 generally induces S-phase cell cycle arrest by inhibiting CDK2 activity [64–66] which, together with cyclin A, is necessary for the progression of cells through the S phase [67]; inactivation of cyclin A/CDK2 complexes prevents progression beyond the S phase checkpoint.

CDK2 is the major downstream target of Cdc25A, which activates the cyclin A/CDK2 complex [68]. Triptolide and simvastatin can induce S-phase arrest by inhibiting Cdc25A [69, 70]. In addition, Chk1, which is regulated by ATR, phosphorylates the Cdc25A protein at S76, which in turn up-regulates ubiquitin-mediated proteolysis of Cdc25A [47, 43]. Chk1 also regulates the S phase checkpoint by promoting proteolysis of Cdc25A, which in turn inhibits CDK2 activity, in response to some anticancer agents, such as infrared ray (IR) and ultraviolet (UV) light [71–73]. Our results demonstrate that NaF decreased cyclin A and CDK2 and increased ATR and Chk1 protein levels and mRNA expression (Figures 8, 10, 18, and 19). However, p-Cdc25A (S76) protein levels increased, while Cdc25A mRNA expression decreased, after NaF treatment (Figure 20). The increases in p-ATR, p-Chk1, and p-Cdc25A protein levels suggest that NaF activates the ATR signal transduction pathways. In addition, downregulation of Cdc25A induced S-phase arrest by inhibiting activation of the cyclin A/CDK2 complex. Cyclin D1, along with its kinase partners CDK4 and CDK6, is a major mitogen-induced regulator of cell cycle progression in the G1 phase. Cyclin E/CDK2 complexes are crucial for the transition from the G1 to the S phase [3]. As shown in Figures 7 and 9, NaF markedly reduced cyclin E and CDK4 protein levels and mRNA expression, thus inhibiting the transition from the G1 to the S phase. The decreases observed in cyclin E, cyclin A, Cdc25A, and CDK2 protein levels and mRNA expression are consistent with a previous report that triptolide induced S phase arrest in multiple myeloma cells by inhibiting cyclin E, cyclin A, Cdc25A, and CDK2 expression and up-regulating p21 and p27 expression [69].

The PCNA protein, a marker of proliferation that plays crucial roles in many cellular processes, is ubiquitously expressed in eukaryotic cells. For example, PCNA is involved in DNA replication, DNA repair, and chromatin assembly and maintenance [74–76]. We found that NaF decreased PCNA protein levels and mRNA expression (Figure 11), suggesting that NaF also inhibits hepatocellular proliferation by down-regulating PCNA expression. These decreases in PCNA protein levels and mRNA expression are consistent with a previous report that fluoride can decrease germ cell counts and damage the male reproductive system by inhibiting PCNA expression [77].

**Figure 21:**
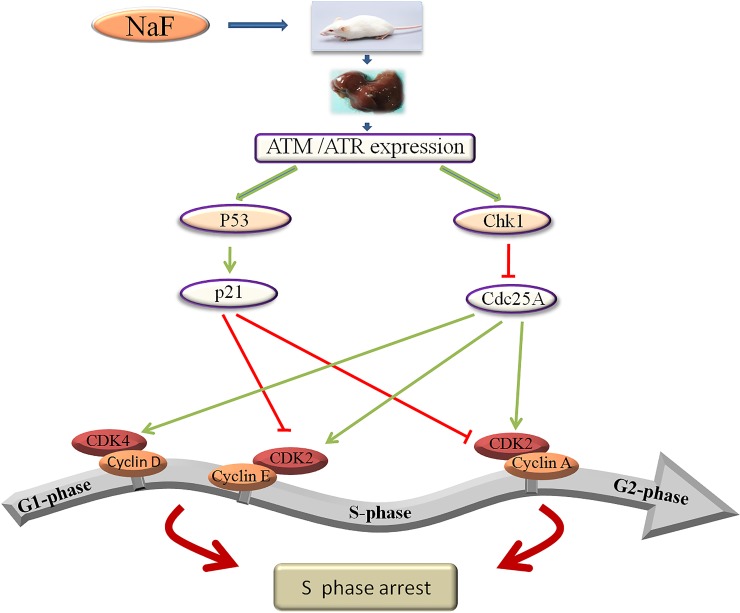
Schematic of NaF-induced S-phase cell cycle arrest in mouse liver NaF can activate two signal transduction pathways: the ATM-p53-p21 and ATR-Chk1-Cdc25A pathways, which cause hepatocellular cell cycle arrest at S-phase.

In conclusion, the results of this study demonstrate that NaF increases ATM, p53, p21, ATR, and Chk1 expression, decreases Cdc25A, cyclin E, cyclin A, CDK2, CDK4, and PCNA expression, activates the ATM-p53-p21 and ATR-Chk1-Cdc25A pathways, and ultimately leads to S-phase arrest in the mouse liver (Figure 21).

## MATERIALS AND METHODS

### Animals and treatments

240 healthy ICR mice were provided by the Experimental Animal Corporation of Dossy in Chengdu, China. Food and water were provided *ad libitum*. After 1 week of acclimatization, mice were divided randomly into four groups (N=60). The control group received intragastric doses of distilled water, while the experimental groups received intragastric doses of 12, 24, or 48 mg/kg NaF (Chengdu Kelong Chemical Co., Ltd., Chengdu, China). NaF was diluted with distilled water. The gavage doses for all four groups were 1 mL/100 g body weight once daily for 42 days. Liver samples were collected from mice on days 21 and 42 of the experiment.

The use of mice and all experimental procedures were approved by the Animal Care and Use Committee, Sichuan Agricultural University.

### Determination of liver growth index

After body weights were recorded, eight mice per group were humanely killed on days 21 and 42 of the experiment. Gross observations and weights were recorded for each liver. Liver growth index (GI) was calculated according to the following formula:

### Pathological observations

Liver samples were taken from eight mice in each group on days 21 and 42 of the experiment and gross observations were recorded. After gross examination, liver samples were fixed in 4% paraformaldehyde solution, dehydrated in ethanol, and embedded in paraffin. Serial slices at 5 μm thickness were prepared and stained with hematoxylin and eosin (H·E) for histopathological examination under a light microscope.

### Flow cytometry cell cycle assay

Eight mice per group were humanely killed on days 21 and 42 of the experiment; liver samples were immediately removed and cut into pieces to form a cell suspension that was filtered through a 300-mesh nylon screen. The cells were washed twice with cold PBS (phosphate buffer solution, pH 7.2-7.4) and then suspended in PBS at a concentration of 1×10^6^ cells/mL using the normal counting method for blood cells. 100 μL portions of the cell suspension were transferred into new 5 mL culture tubes. The cells were then incubated for 30 min at room temperature in the dark with 0.25% Triton X-100 and 5 μL propidium iodide (PI) (BD, Cat. No.51–66211E, USA). Cells were resuspended in 0.5 mL PBS and run on a BD FACS Calibur flow cytometer. The results were analyzed using Mod Fit LT for Mac V3.0.

### Cell cycle regulatory protein expression by western blot

On days 21 and 42 of the experiment, liver samples were collected from eight mice per group and the expression of cell cycle regulatory proteins was examined by western blot. The liver was homogenized and proteins were extracted with RIPA lysis buffer and kept in Laemmli loading buffer. Protein samples were resolved on SDS-PAGE gels (5%–15%) and transferred to nitrocellulose filter membranes. Membranes were blocked with 5% fat-free milk for 1h and incubated with primary antibodies overnight at 4°C. The primary antibodies were cyclin D/E/B/A, CDK1/2/4, p-ATM, p-p53, p21, p-ATR, p-Chk1, and p-Cdc25A (Table 1). The membranes were then washed with PBS-Tween, incubated with biotin-conjugated secondary antibodies (Santa Cruz, USA) for 1h, and washed again with PBS-Tween. Blots were visualized by ECL™ (Bio-Rad, Hercules, CA, USA) and X-ray film. Statistical analysis of protein expression was performed using ImageJ2x software.

**Table 1:** Antibodies used in western blot

Name	Company	Cat#	dilution
cyclin E	Abcam, China	ab52189	1/1000
Cyclin A	Abcam, China	ab181591	1/1000
Cyclin D	Abcam, China	ab134175	1/1000
Cyclin B	Abcam, China	ab181593	1/1000
CDK1	Abcam, China	ab32384	1/1000
CDK2	Abcam, China	ab76146	1/1000
CDK4	Santa Cruz, China	sc260	1/50
p-ATR	Gene Tex, China	GTX128145	1/500
p-Chk1	Cell Signaling, China	12302T	1/100
p-Cdc25A	Santa Cruz, China	sc101655	1/50
p-ATM	Biolegend, China	651201	1/400
p-p53	Cell Signaling, China	9284T	1/1000
p21 PCNA	Boster, China Abcam, China	BA0271 Ab92552	1/100 1/1000

### Cell cycle regulatory molecule mRNA expression by quantitative real-time polymerase chain reaction (qRT-PCR)

On days 21 and 42 of the experiment, liver samples were collected from eight mice per group and stored in liquid nitrogen. They were then homogenized in liquid nitrogen using a mortar and pestle. Total RNA was extracted from frozen liver powders using RNAiso plus (9108/9109, Takara, China) following the manufacturer's instructions. cDNA was then synthesized using a PrimScript™ RT reagent Kit (RR047A, Takara, China) according to the manufacturer's instructions. The cDNA product was used as a template for qRT-PCR analysis. Specific oligonucleotide primers were designed and synthesized by Sangon Biotech (Shanghai, China; Table 2) according to the *Mus musculus* sequences. All qRT-PCR was performed on a Model C1000 Thermal Cycler (Bio Rad, USA) using the SYBR® Premix Ex Taq™Ⅱ system (RR820A, Takara, China) according to the standard protocols.

**Table 2:** Sequence of primers used in qRT-PCR

Gene symbol	Accession number	Primer	Primer sequence(5’-3’)	Product size
cyclin E	NM_007633	Forward	GTTACAGATGGCGCTTGCTC	104
		Reverse	AGCCAGGACACAATGGTCAG	
Cyclin A	NM_009828	Forward	CTTGTAGGCACGGCTGCTAT	451
		Reverse	CATGTTGTGGCGCTTTGAGG	
Cyclin D	NM_007631	Forward	AAGTGTGACCCGGACTGC	174
		Reverse	GATGTCCACATCTCGCACG	
Cyclin B	NM_172301	Forward	AGCGAAGAGCTACAGGCAAG	141
		Reverse	CTCAGGCTCAGCAAGTTCCA	
CDK1	NM_007659	Forward	TCGGCTCGTTACTCCACTC	154
		Reverse	GCCACACTTCGTTGTTAGG	
CDK2	NM_016756	Forward	TTGGAGTCCCTGTCCGAACT	142
		Reverse	CGGGTCACCATTTCAGCAAAG	
CDK4	NM_009870	Forward	CAATGTTGTACGGCTGATGG	120
		Reverse	GGAGGTGCTTTGTCCAGGTA	
Chk1	NM_007691.5	Forward	GCAAACTTTGGGAGAAGGTGC	103
		Reverse	TATGGCCCGCTTCATGTCTAC	
Cdc25A	NM_007658	Forward	AGAACCCTATTGTGCCTACTG	124
		Reverse	TACTCATTGCCGAGCCTATC	
ATM	NM_007499.2	Forward	CCTTCCCACTCCAGAAACAG	123
		Reverse	CTCCGCATAACTTCCATCGT	
p53	NM_011640.3	Forward	AGAGACCGCCGTACAGAAGA	227
		Reverse	GCATGGGCATCCTTTAACTC	
p21	U24173.1	Forward	CAAAGTGTGCCGTTGTCTCTT	111
		Reverse	TCAAAGTTCCACCGTTCTCG	
PCNA	NM_011045	Forward	ATCCCAGAACAGGAGTACAGC	92
		Reverse	ACAGCATCTCCAATGTGGCT	
β-actin	NM_007393	Forward	GCTGTGCTATGTTGCTCTAG	117
		Reverse	CGCTCGTTGCCAATAGTG	

Mice β-actin expression was detected as an internal reference housekeeping gene. Gene expression from control group samples on days 21 and 42 of the experiment was used to calibrate gene expression in samples from the experimental groups. The 2^-ΔΔCT^ method was used to analyze data from the qRT-PCR experiments [78].

### Statistical analysis

One-way analysis of variance (ANOVA) procedure in SPSS 18.0 software was carried out to analyze all the data. All the results were expressed as mean ± standard deviation. *p* < 0.05 was significant, and *p* < 0.01 was markedly significant.
